# Quantitative Proteomics at Early Stages of the Symbiotic Interaction Between *Oryza sativa* and *Nostoc punctiforme* Reveals Novel Proteins Involved in the Symbiotic Crosstalk

**DOI:** 10.1093/pcp/pcac043

**Published:** 2022-04-04

**Authors:** Consolación Álvarez, Manuel Brenes-Álvarez, Fernando P Molina-Heredia, Vicente Mariscal

**Affiliations:** Instituto de Bioquímica Vegetal y Fotosíntesis, Consejo Superior de Investigaciones Científicas and Universidad de Sevilla, cicCartuja, Américo Vespucio 49, Seville 41092, Spain; Instituto de Bioquímica Vegetal y Fotosíntesis, Consejo Superior de Investigaciones Científicas and Universidad de Sevilla, cicCartuja, Américo Vespucio 49, Seville 41092, Spain; Instituto de Bioquímica Vegetal y Fotosíntesis, Consejo Superior de Investigaciones Científicas and Universidad de Sevilla, cicCartuja, Américo Vespucio 49, Seville 41092, Spain; Departamento de Bioquímica Vegetal y Biología Molecular, Facultad de Biología, Universidad de Sevilla, Avda. Reina Mercedes s/n, Seville 41012, Spain; Instituto de Bioquímica Vegetal y Fotosíntesis, Consejo Superior de Investigaciones Científicas and Universidad de Sevilla, cicCartuja, Américo Vespucio 49, Seville 41092, Spain

**Keywords:** Cyanobacteria, Differential proteomic, Nitrogen, Rice, Symbiosis

## Abstract

Symbiosis between cyanobacteria and plants is considered pivotal for biological nitrogen deposition in terrestrial ecosystems. Despite extensive knowledge of the ecology of plant–cyanobacterium symbioses, little is known about the molecular mechanisms involved in recognition between partners. Here, we conducted a quantitative sequential window acquisition of all theoretical fragment ion spectra mass spectrometry pipeline to analyze protein changes in *Oryza sativa* and *Nostoc punctiforme* during early events of symbiosis. We found differentially expressed proteins in both organisms linked to several biological functions, including signal transduction, adhesion, defense-related proteins and cell wall modification. In *N. punctiforme* we found increased expression of 62 proteins that have been previously described in other *Nostoc*–plant symbioses, reinforcing the robustness of our study. Our findings reveal new proteins activated in the early stages of the *Nostoc*–*Oryza* symbiosis that might be important for the recognition between the plant and the host. *Oryza* mutants in genes in the common symbiosis signaling pathway (CSSP) show reduced colonization efficiency, providing first insights on the involvement of the CSSP for the accommodation of *N. punctiforme* inside the plant cells. This information may have long-term implications for a greater understanding of the symbiotic interaction between *Nostoc* and land plants.

## Introduction

The use of symbiotic N_2_-fixing microorganisms to explore new sustainable agricultural practices of cereals is a key strategy in the alleviation of adverse environmental consequences of the synthetic fertilizers. This is of special relevance in rice, the primary cereal produced and a staple food for almost half of the global human population (http://www.fao.org/faostat/en/#data/QC, November 11, 2021, date last accessed). Nitrogen is generally the most limiting nutrient for rice production, since synthetic N fertilizers used for rice cultivation are transformed to other nitrogen sources and are largely lost from flooded soil by nitrate leaching and nitrous oxide (N_2_O) emission (Ishii et al. 2011, Liu et al. 2019a). A variety of beneficial N_2_-fixing cyanobacteria that naturally occur in rice paddies have been shown to enhance plant growth (Vaishampayan et al. 2001, Iniesta-Pallarés et al. 2021). However, despite the beneficial effects of cyanobacterial nitrogen fixation in these terrestrial ecosystems, little is known about both the intra-partner chemical and molecular signaling mechanisms and the metabolic adaptations underlying the symbiotic process.

*Nostoc punctiforme* is a versatile N_2_-fixing cyanobacterium that has been extensively used as a model organism to study plant–cyanobacteria symbiosis (Meeks et al. 2001, Meeks and Elhai 2002). It exists as a free-living organism or in symbiosis, with representatives from the four major phylogenetic divisions of terrestrial plants, thus reflecting high diversity and low host specificity in its symbiotic interactions (Svenning et al. 2005, Adams et al. 2013, Warshan et al. 2018, Álvarez et al. 2020). *Nostoc* has differing symbiotic associations with host plants. For example, in liverworts and hornworts it grows in specialized compartments, but do not colonize the plant cells; however, it colonizes intracellularly stem glands of *Gunnera* sp. or root cells of *Triticum vulgare* and *Oryza sativa* where it provides fixed nitrogen to the host plant (Johansson and Bergman 1992, Gantar et al. 1993, Álvarez et al. 2020).

The symbiotic interaction of *Nostoc* with plants comprises two phases: an initial phase of recognition, involving chemical signaling between partners, and a later phase of physical contact, when the cyanobacterium begins to colonize and intimately associate with the plant. At the recognition phase, chemical signaling in the plant partner involves the secretion of flavonoids (Cohen and Yamasaki 2000, Cohen et al. 2002) and diacylglycerols, triggering hormogonium differentiation in *Nostoc* (Hashidoko et al. 2019). Hormogonia are motile cyanobacterial infection units (Meeks and Elhai 2002) that are chemoattracted to plants by soluble sugars (Nilsson et al. 2006, Khamar et al. 2010). Symbiotic *Nostoc* strains produce a variety of small peptide-like substances that are tightly connected to their free-living state (Liaimer et al. 2016; Wharshan et al. 2018). However, the cyanobacterial signals involved in plant recognition remain unknown. The second phase involves metabolic and structural changes in both partners to accommodate a symbiotic lifestyle.

Several genes have been predicted to be differentially regulated during a symbiotic association with a host plant (Warshan et al. 2017, 2018) and verified by transcriptomic and proteomic analyses (Ekman et al. 2006, Campbell et al. 2008, Warshan et al. 2017). Early genes involved in the pre-symbiotic process are related to signal transduction, oxidative stress response, chemotaxis or motility. Late genes, required once the symbiosis has been stabilized, are involved in the transport of nutrients, such as phosphate, amino acids, sugars and ammonium. It has been proposed that, in symbiosis, the cyanobacterium shifts from a photoautotrophic to a heterotrophic lifestyle, relying on the carbon provided by the host to sustain N_2_ fixation (Meeks and Elhai 2002). Relatively little is known about the changes in plant hosts in response to the cyanobacterium in the early phase of interaction, with only very recent studies using RNA-seq showing upregulation of receptor kinases and stress response genes in *Anthoceros punctatus* and *Arabidopsis thaliana* responding to *Nostoc* interactions (Li et al. 2020, Belton et al. 2021).

Molecular genetic studies have revealed that plants engaging intracellular microbial symbioses, such as Leguminosae–*Rhizobium, Parasponia* (non-legume)–*Rhizobium*, actinorhizal plants–*Frankia* and arbuscular mycorrhizal (AM) symbioses with fungi of the Glomeromycotina clade, share a genetic toolkit that is necessary for symbiont accommodation (Geurts et al. 2016, Radhakrishnan et al. 2020, Baldo and Werren 2021). These signaling pathways comprise a well-conserved network known as the ‘common symbiosis signalling pathway’ (CSSP) (Oldroyd 2013, Radhakrishnan et al. 2020). This symbiotic signaling process is initiated when the host secretes factors, known as Nod factors in *Rhizobium* and Myc factors in AM fungi, upon sensing flavonoids produced by compatible plant partners. Nod and Myc factors are primarily sensed by a set of LysM-Receptor-Like Kinases, including Nod factor receptors 1 and 5 and SymRK, activating the production of an unknown secondary messenger that activates POLLUX and CASTOR cation channels. These channels produce nuclear membrane repolarization that initiates calcium spiking (Kim et al. 2019). In the nucleus, calmodulin-dependent protein kinase (CCaMK) decodes calcium oscillations, which interact and phosphorylate CYCLOPS, a transcriptional activator that induces a transcriptional cascade leading to symbiosis (Parniske 2008, Mbengue et al. 2020). Genetic and phylogenetic studies have revealed that CSSP is required in endosymbiotic associations in which microbes colonize living plant cells, being conserved in all intracellular symbioses-forming land plant lineages (Radhakrishnan et al. 2020). Analysis of homozygous mutant lines of *O. sativa CCaMK, CYCLOPS, POLLUX* and *CASTOR* genes has revealed that CSSP is essential for *O. sativa*–AM fungi symbiosis (Gutjahr et al. 2008).

In contrast to the extensive knowledge of the signaling mechanisms in *Rhizobium*–legume symbioses and AM fungi–plant symbioses, information about signaling networks involved in *Nostoc* symbioses is scarce. In the present study, we evaluated proteomic changes that occur in *O. sativa* and *N. punctiforme* in a pre-symbiotic stage before the colonization step. The changes were determined by sequential window acquisition of all theoretical mass spectra (SWATH-MS) (Huang et al. 2015, Liu et al. 2019b). We report a new pipeline for simultaneous proteomic analysis in both organisms, suggesting a valuable approach that may be applied to the study of other plant–microbe interactions. We have identified and quantified 1,397 and 1,555 proteins from *N. punctiforme* and *O. sativa,* respectively, providing a valuable analysis of the metabolic routes activated in each organism at early stages of interaction. While some of the proteins have previously been associated with plant–microbe interactions, other proteins are newly described in this context. Furthermore, impaired *N. punctiforme* colonization in different *O. sativa* CSSP mutants revealed the involvement of this ancient signaling pathway in the symbiosis between *N. punctiforme* and *O. sativa.*

## Results

### Timing the symbiosis between *O. sativa* and *N. punctiforme*

**Fig. 1:**
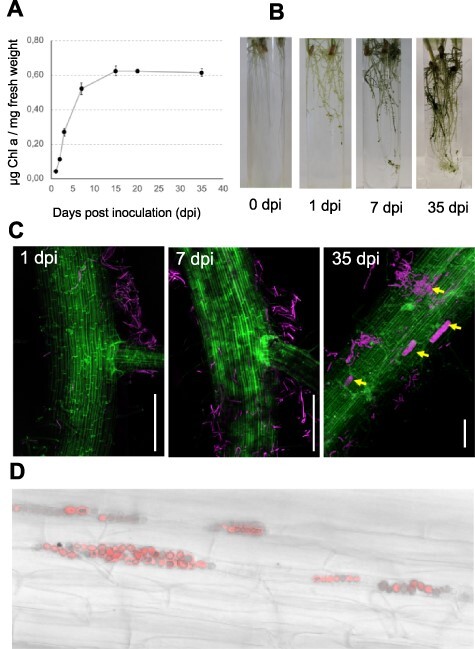
Association of *N. punctiforme* with *O. sativa* sp. Indica roots. (A) Green appearance of *O. sativa* roots inoculated with *N. punctiforme* at 0, 1, 7 and 35 dpi. (B) Cyanobacterial association with rice roots was quantified as μg chlorophyll *a* (Chl *a*)· mg^−1^ root weight. The values are the means ± standard error of the mean from t triplicate experiments. (C) *O. sativa* roots inoculated with *N. punctiforme* at 1, 7 and 35 dpi were visualized by confocal microscopy with Z stacks generated from 90 to 130 frames. Arrows indicate the plant cells colonized. Scale bar: 100 µm. Autofluorescence from the plant cell walls is colored green, while cyanobacterial chlorophyll autofluorescence is colored magenta. (D) Closed view of a plant root colonized with *N. punctiforme at* 35 dpi. Cyanobacterial filaments (in red) are enclosed inside the plant epidermal cells. Merged images of red autofluorescence and bright field are shown.

The different stages of symbiosis between *N. punctiforme* and *O. sativa* were monitored from 1 to 35 days post inoculation (dpi) by inoculating *O. sativa* hydroponic cultures with *N. punctiforme* hormogonia grown in BG11_0_ (cyanobacterial growth medium free of combined nitrogen). Interaction between partners was detected by optical visualization (**Fig. 1A**) and quantified by measuring the chlorophyll content of the roots, indicative of the amount of *N. punctiforme* attached to the roots (**Fig. 1B**). Microscopic inspection by means of confocal microscopy was also addressed (**Fig. 1C**). We detected the first phase of chemical signaling at 1 dpi, in which *N. punctiforme* hormogonia were attracted to the rice roots and became entangled in the root hairs (**Fig. 1C**). A second phase involving physical contact occurred from 1 to 15 dpi. Chlorophyll of the rice roots revealed increased growth of the cyanobacterium on the roots surface (**Fig. 1B**), but with any signs of plant colonization (**Fig. 1C**). Fifteen days after co-culture, the amount of chlorophyll in the roots reached a plateau (**Fig. 1A**), and a third phase consisting of plant intracellular colonization began with entirely colonized plant trichoblasts and atrichoblasts evident at 35 dpi (**Fig. 1C, D**), with a significant increase in the length and fresh weight of plants (**Supplementary Fig. S1**). Since we aimed to elucidate the signaling mechanisms involved in the pre-symbiotic process, we selected 1 dpi and 7 dpi for subsequent analyses. At this stage, any event of colonization was observed.

### Simultaneous quantitative proteomics of *N. punctiforme* and *O. sativa*

**Fig. 2:**
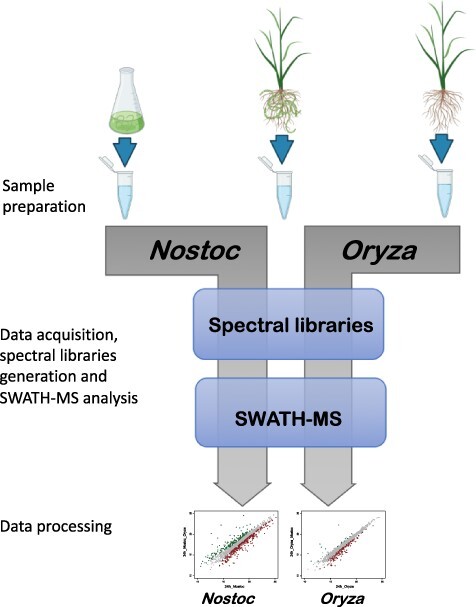
Overview of the schematic workflow used for quantitative proteomic analysis in *N. punctiforme* and *O. sativa*. The first step includes sample preparation and data acquisition by liquid chromatography-tandem mass spectrometry for generation of species-specific spectral libraries. The second step determines relative quantification of protein changes by SWATH-MS analysis. The subsequent data normalization was made with LIMMA R package to extract changes in protein abundance.

Pre-symbiotic quantitative proteomic analysis of the early steps of recognition between *N. punctiforme* and *O. sativa* was performed at 1 dpi and 7 dpi (as described in Materials and Methods). As we extracted proteins from both organisms at the same time, we generated species-specific spectral libraries that were used for the identification and quantification of protein changes in each organism by SWATH-MS analysis (**Fig. 2**). At both time points, 1,397 and 1,555 proteins from *N. punctiforme* and *O. sativa,* respectively, were identified and quantified.

**Fig. 3:**
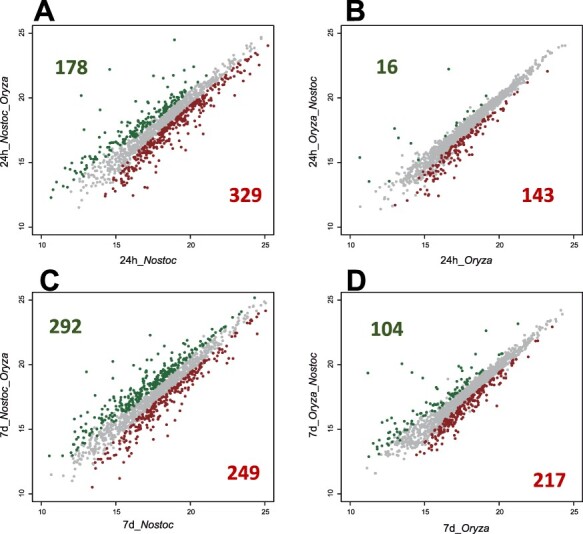
Global protein expression changes in *N. punctiforme* and *O. sativa* during early symbiosis. (A, C), Proteins significantly activated and repressed in *N. punctiforme* in response to *O. sativa* at 1 and 7 d, respectively. (B, D) Proteins significantly activated and repressed in *O. sativa* in response to *N. punctiforme* at 1 and 7 d, respectively. Significantly increased and decreased protein expression, analyzed by LIMMA R package, is shown in green and red, respectively (fold change >1.5 or <0.66667, and adjusted *P*-value < 0.05).

The initial characterization and verification of our experimental design involved an examination of samples using principal component analysis (PCA). The duration of interaction and sample type were the major determinants for the data generated, with no significant batch effect or confounding factors. Samples segregated into well-separated groups, and biological replicates clustered tightly together (**Supplementary Fig. S2**). No outlier values were detected; therefore, all replicates were used for further analyses. The similarities and differences between *O. sativa* and *N. punctiforme* were analyzed by pairwise comparisons (Supplementary data File 1). The abundance of 666 proteins (159 plant and 507 cyanobacterial) and 862 proteins (321 plant and 541 cyanobacterial) was significantly affected (*P* ≤ 0.05, fold change ≥1.5 or ≤0.67) at 1 dpi and 7 dpi, respectively, relative to non-inoculated samples (**Fig. 3**).

**Fig. 4:**
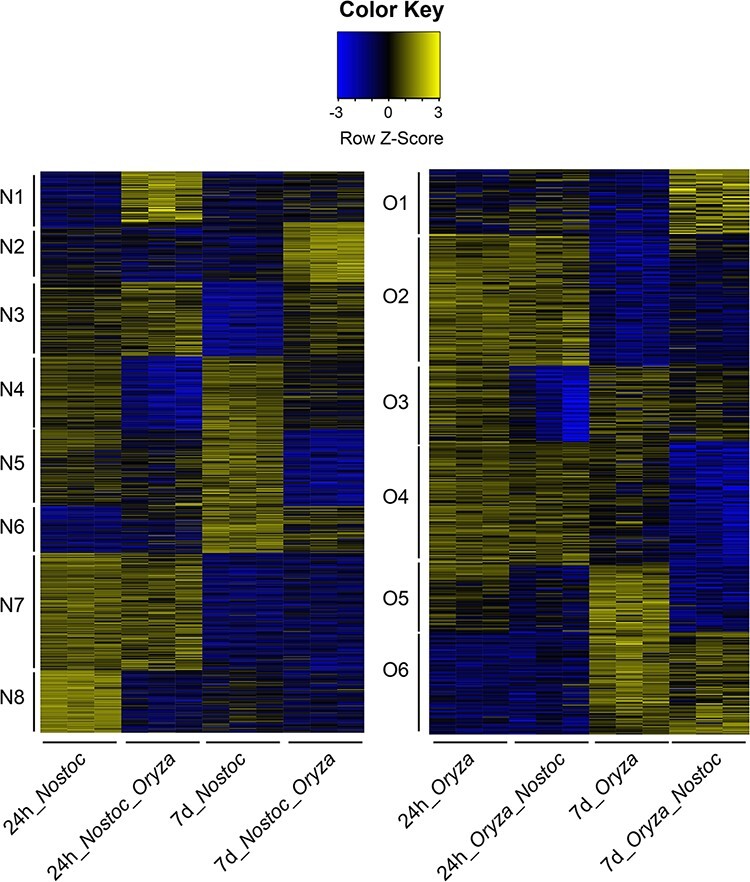
Heat map profiles of differentially expressed proteins in *N. punctiforme* and *O. sativa.* Differentially expressed proteins were clustered into eight and six groups for *N. punctiforme* (N1 to N8) and *O. sativa* (O1 to O6), respectively, based on their expression profile (Z-score by row) and Mfuzz analysis. In each condition, the average expression of each of the three technical replicates is shown. Scale bar: blue, downregulated; yellow, upregulated.

In order to ascertain groups of proteins with the same regulation, a clustering analysis using Mfuzz package was carried out in both organisms (Kumar and Futschik 2007). Clustering analysis revealed eight protein clusters with proteins with the same regulation in both organisms in *N. punctiforme* and six clusters in *O. sativa* with at least 80% confidence (**Supplementary Figs. S3, S4**). A heat map representation of the proteins in each cluster illustrating the consistency of clustering and between-individual replicates of the treatments is shown in **Fig. 4**. We found (i) clusters of proteins upregulated in co-culture at 1 dpi (N1) and 7 dpi (N2, N3 and O1), (ii) clusters of proteins downregulated in co-culture at 1 dpi (N4, N8 and O3) and 7 dpi (N5, O4 and O5), (iii) clusters of proteins repressed at 7 dpi, independently of the presence of the partner (N7 and O2) and (iv) clusters of proteins induced at 7 dpi, independently of the presence of the partner (N6 and O6). Proteins belonging to each cluster can be found in Supplementary data File 1.

### Proteins differentially accumulated in *N. punctiforme* and *O. sativa* during symbiosis

**Fig. 5:**
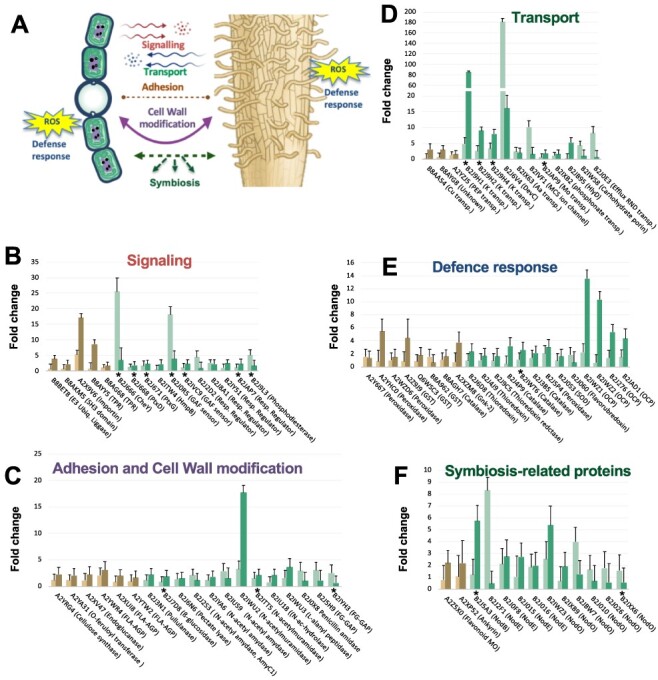
Selected metabolic group of proteins significantly activated in *O. sativa* and *N. punctiforme* at 1- and 7 dpi. (A) An overview of the main functions with significant changes when both organisms were in contact. (B–F) Fold change of proteins in each of the selected metabolic groups at 1 and 7 dpi. The fold change was calculated with respect to the corresponding control without the partner in the same condition. All the proteins show a significant increase in expression with respect to the control (*P*-value < 0.05). Asterisk denotes proteins found in other *Nostoc*–plant symbioses.

In *O. sativa*, 104 proteins were significantly increased in abundance in the presence of *N. punctiforme* (mostly clustered in O1 and O2). Most of the changes in the plant were observed at 7 dpi, with only 16 proteins induced at 1 dpi. In *N. punctiforme*, 178 proteins were significantly increased in abundance at 1 dpi (clustered in N1) and 292 proteins were induced at 7 dpi (clustered in N2 and N3). Of these, 81 proteins were induced at both 1 dpi and 7 dpi. The differentially accumulated proteins were manually organized into metabolic groups shown in Supplementary data File 1 (with selected proteins shown in **Fig. 5**) to allow analysis of their function in relation with their regulation. Among others, signaling, adhesion and cell wall modification, transport, defense response and symbiosis-related proteins were analyzed in detail (**Fig. 5A**).

#### Signaling.

In *O. sativa*, two response regulators (B8AYY5 and B8AG68), a SH3 domain-containing protein (B8AKM5) and an importin-α (A2X9V6), showed increased abundance in response to *N. punctiforme* (**Fig. 5**). In *Lotus japonicus,* rhizobial infection enhances transcript level of the SIE3, encoding an E3 Ubiquitin ligase interacting with SymRK (Yuan et al. 2012). An E3 ubiquitin ligase (B8BET8), increased 3.93\ times at 7 dpi in co-culture, was also identified in our proteomic analysis (**Fig. 5B**).

In *N. punctiforme* 11 signaling proteins showed increased abundance in response to the plant (**Fig. 5B**), with seven of these previously identified in other *Nostoc* symbioses with *Pleurozium schreberi, A. punctatus* and *Gunnera manicata* (Ekman et al. 2006, Campbell et al. 2008, Warshan et al. 2017). Three proteins were from a cluster of genes involved in chemotaxis and phototaxis (Campbell et al. 2015); these included CheY (B2J666), with an increased abundance of 25.51 times at 1 dpi in the presence of the plant, and two methyl-accepting chemotaxis proteins (B2J668 and B2J671). The abundance of the response regulator receiver HmpB (B2ITW4) and two GAF sensor signal transduction histidine kinases (B2J0R5 and B2IVC9) was also found to be increased. Other proteins not previously described included four response regulator receiver proteins (B2J2D2, B2J8A1, B2IY51 and B2JAP7) and a putative cGMP-specific 3ʹ,5ʹ-cyclic phosphodiesterase (B2J9L3).

#### Cell adhesion and cell wall modification.

Proteins involved in cell adhesion and cell wall modification had a higher upregulation mainly at 7 dpi, consistent with the physical contact that is observed at this time (**Fig. 5C**). In *O. sativa*, proteins that were significantly increased in abundance included three fasciclin-like arabinogalactan proteins (FLAs) (A2YWR4, A2XUI8 and A2YTW2) (**Fig. 5C**), a subclass of arabinogalactan proteins (AGPs), regulating functions linked to cell wall biosynthesis, cell adhesion and signaling (Wu et al. 2020). Proteins involved in remodeling the plant cell wall were also differentially accumulated in *O. sativa* in contact with *N. punctiforme* (**Fig. 5C**). Thus, a cellulose synthase (A2YRG4), a putative endoglucanase (A2XV47) and an omega-hydroxypalmitate *O*-feruloyl transferase (A2YA31), involved in the synthesis of the suberin polymer (Gou et al. 2009), were differentially accumulated.

In *N. punctiforme,* two cell adhesion FG-GAP repeat proteins (B2J5H9 and B2IYH3) were identified. FG-GAP proteins are involved in chemical and physical contacts in the *N. punctiforme*–*P. schreberi* symbiosis (Warshan et al. 2017). Proteins involved in cyanobacterial peptidoglycan recycling and modulation were also increased in abundance in contact with the plant (**Fig. 5C**). They included three *N*-acetylmuramoyl-L-alanine amidases (B2J2S3, B2IYA6 and B2IUS9), two *N*-acetylmuramidases (B2IWU2 and B2ITT5), an *N*-acetylmuramic acid 6-phosphate hydrolase (B2IU18), an L-alanyl-D-glutamate peptidase (B2IWU3) and a penicillin amidase (B2J0X8). Accumulation of these peptidoglycan-modifying enzymes is indicative of physiological changes in the envelope of the cyanobacterium, which may be related to the attachment to the plant surface and adaptation of the symbiotic lifestyle. Intracellular accommodation of the microbial partner in the plant cells requires mechanisms to overcome the plant cell wall barrier. In *N. punctiforme*, proteins differentially accumulated included the cell-wall-modifying enzymes pullulanase (1,4-alpha-glucan branching enzyme GlgB, B2J3N1), a putative β-glucosidase (B2J7D8) and a pectate lyase (B2J6N6). Induction of pectate lyase was previously shown in the exoproteome of *N. punctiforme* in contact with *P. schreberi* (Warshan et al. 2017). The induction of these enzymes might imply a remodeling of the cell plant wall in the presence of the host, similar to that previously reported in the *Medicago truncatula*–*Gigaspora margarita* symbiosis (Siciliano et al. 2007).

#### Transport.

Transfer of ions and metabolites through membrane-bound transport proteins is key in nutritional symbiotic interactions (Roy et al. 2020). In *O. sativa*, three transport systems were increased in abundance at 7 d in the presence of the cyanobacterium. They were a Ctr-type copper transporter (B8AA54), a triose-phosphate transporter (A2YZJ5) and an unknown transporter from the transmembrane 9 superfamily (TM9SF) (B8AYG8).

In *N. punctiforme*, 11 transporters involved in the uptake and extrusion of substances were activated in the presence of *Oryza*. DevC (B2J6V4), the periplasmic membrane fusion component of polysaccharide ABC exporter, is one of the proteins with the highest abundance level in our proteomic analysis in *N. punctiforme* (181.04 times at 1 dpi). In *Nostoc* sp. PCC 7120, this exporter is essential for cyanobacterial envelope formation (Shvarev et al. 2019). Multidrug efflux systems not only cause resistance against antibiotics and toxic compounds but also mediate successful host colonization by certain plant-associated bacteria (Lindemann et al. 2010). An Resistance-Nodulation-Division (RND) efflux transporter (B2J0E3) showed increased abundance at 1 dpi in the presence of *Oryza*. We also found differential accumulation in uptake transport systems, including a periplasmic component of a polar amino acid ABC transporter (B2IX63), three components of a potassium transporter (B2J9H1, B2J9H2 and B2J9H4), the periplasmic component of a phosphonate ABC transporter (B2IXB2), a molybdenum ABC transporter (B2JAP9) and a MscS mechanosensitive ion channel (B2IVF1). A carbohydrate-selective porin OrpB (B2IWS8) was also differentially accumulated at 1 dpi in response to *Oryza* (**Fig. 5D**).

#### Defense response and oxidative stress.

Both pathogenic and symbiotic microbes produce similar microbe-associated molecular patterns (MAMPs) that are, at early stages, indistinguishable to the plant (Zhao and Qi 2008). Thus, these MAMPs, regardless of their origin, activate defense responses via similar molecular signaling pathways. In the physical contact phase (7 dpi), both organisms upregulated several proteins involved in the defense response (**Fig. 5E**). In *O. sativa*, three class III peroxidases (A2Y667, A2YHC0 and A2WZD6), three glutathione S-transferases (A2Z9J9, Q6WSC3 and B8A962), catalase (B8AGH7) and a Ginkbilobin-2-homologous domain-containing protein (A2XZM8) were differentially accumulated. Class III plant peroxidases are well-known proteins induced during the host plant defense that have been detected in other plant–microbe symbioses (Guimil et al. 2005, Gutjahr et al. 2008, 2015).

In *N. punctiforme*, two thioredoxins (B2J6D8 and B2J4J9), thioredoxin reductase (B2J9C5), three catalases (B2J4P9, B2IWT6 and B2J3B5), peroxidase (B2J5P4), superoxide dismutase (B2J0S3), flavorubredoxin (B2J060) and four proteins similar to the N-terminal domains (NTD) of the photoactive orange carotenoid protein (B2IWZ1, B2IWZ2, B2J276 and B2JAD1) showed increased abundance in response to the plant partner. NTD-like proteins are involved in photoprotection and the response to oxidative stress (López-Igual et al. 2016), while it is well known that catalases are essential for the establishment of plant–microbe symbioses (Jamet et al. 2003). Peroxidase B2IWT6 and catalase B2J3B5 are expressed in *N. punctiforme* in symbiosis with *P. schreberi* (Warshan et al. 2017).

#### Symbiosis-related proteins.

Flavonoids are crucial signaling molecules involved both in defense responses to pathogens and symbiosis with beneficial microorganisms (Desta et al. 2016, Liu and Murray 2016). In *O. sativa*, a putative flavonoid 3ʹ-monooxygenase (A2Z5X0) involved in eriodyctiol biosynthesis from naringenin showed increased abundance in response to *N. punctiforme* (**Fig. 5F**). Naringenin is exuded by some legume roots and acts as an inducer of *nod* genes in *Rhizobium* (Novák et al. 2002).

In *N. punctiforme*, we found differential abundances of a putative polysaccharide deacetylase similar to NodB (B2J5A3) and four beta-ketoacyl synthases (B2J2F1, B2J0F8, B2J015 and B2J016) similar to NodE from *Rhizobium tropici*. Additionally, we found six putative Ca^2+^-binding proteins with homology to NodO (B2IWZ3, B2IXB9, B2JBW7, B2J010, B2J026 and B2IXX6) required for subsequent infection thread development (Walker and Downie 2020).

### The *O. sativa* CSSP is required for *N. punctiforme* colonization

**Fig. 6:**
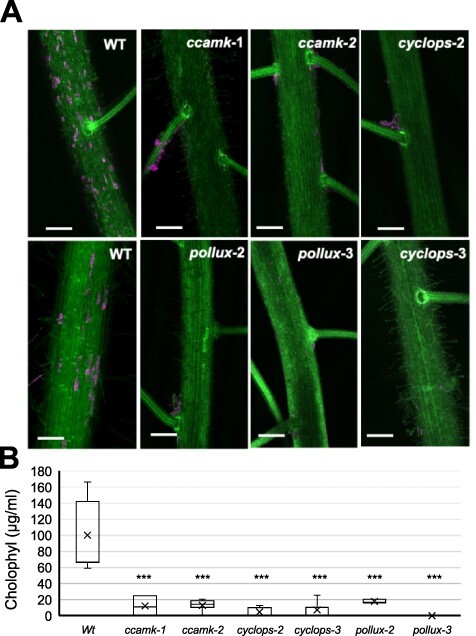
Symbiotic phenotypes of *O. sativa* CSSP mutants. (A) *Oryza sativa* roots inoculated with *N. punctiforme* at 35 dpi were visualized by confocal microscopy with Z stacks generated from 90 to 130 frames. Autofluorescence from the plant cell walls is colored green, while cyanobacterial chlorophyll autofluorescence is colored magenta; scale bar: 200 µm. (B) Number of *O. sativa* root cells that were colonized by *N. punctiforme* in different CSSP mutants in each image. Each data point depicts counting from a single image, with a total of 16–21 images counted from two different colonization assays. Student’s t-test indicated that the differences between the wild type and all the mutants were significant (*P* < 10^–3^).

CSSP is an ancestral signaling pathway conserved in plant lineages forming intracellular symbioses (Radhakrishnan et al. 2020). Genes encoding the components of the CSSP are present in the genome of *O. sativa*, which have been found to be essential for AM fungi infection (Gutjahr et al. 2008). The induction in *N. punctiforme* in response to the plant of proteins with homology to NodB and NodE prompted us to study whether the CSSP is involved in the *Nostoc*–*Oryza* symbiotic interaction. Thus, to test if the CSSP is involved in the *N. punctiforme*–*O. sativa* symbiosis, we performed colonization assays with three *O. sativa* CSSP mutants. These included two homozygous mutant lines for CYCLOPS and POLLUX, the calcium channels that play a role in Ca^2+^ spiking, and two homozygous mutant lines for CCaMK, the calcium/calmodulin-dependent protein kinase that acts downstream of Ca^2+^ spiking (Gutjahr et al. 2008). For this assay, *N. punctiforme* hormogonia were inoculated onto the roots of the mutant plants, and colonization was evaluated by confocal microscopy at 35 dpi. Colonization was severely impaired in all mutants tested, with only a few plant colonization events found sporadically (**Fig. 6**). In order to corroborate the low level of colonization in these mutants, we quantified cyanobacterial biomass from surface-sterilized roots (**Fig. 6B**). We found a significant decrease in the amount of intracellular cyanobacterial biomass. These phenotypes deficient in intracellular symbiosis support the crucial role of the CSSP in *O. sativa*–*N. punctiforme* symbiosis.

## Discussion

Rice is a model system for crop improvement and food security, with most proteomic studies focusing on the response of rice to diverse abiotic and biotic stresses including salt, temperature, nutrients, rice–pathogen interactions and beneficial rice–microbe interactions (Kim et al. 2014, Roth et al. 2018). *Nostoc punctiforme* is a model organism that has been extensively used to study symbiosis with plants (Meeks and Elhai 2002, Adams et al. 2013). However, to the best of our knowledge, symbiosis between cereals and N_2_-fixing cyanobacteria have never been studied at a molecular level, with this being the first study showing proteomic changes in response to symbiotic interactions between *N. punctiforme* and *O*. sativa. This work highlights the pre-symbiotic phases of chemical signaling and physical contact between *N. punctiforme* and *O. sativa* as being critical for the success of the symbiotic process. We are aware that, during this pre-symbiotic stage, co-culture might induce differential growth in both organisms, promoting developmental changes. However, at this stage we did not perceive any differential growth of the organisms in co-culture, with respect to the controls, that might contribute to differential protein accumulation. We have identified and quantified protein changes in both organisms simultaneously, thus facilitating the study of the *N. punctiforme*–*O. sativa* symbiosis in the host microenvironment. In this study, 1,238 differentially expressed proteins have been identified in the early symbiotic phases between *N. punctiforme* and *O. sativa,* providing valued information of the crosstalk between the organisms at the first phase of contact.

In *N. punctiforme*, 389 proteins were differentially accumulated in the presence of *O. sativa*. At least 67 proteins were previously described in other *Nostoc*–plant symbioses, reinforcing the robustness of our study (Supplementary data File 1). Fifty-four proteins differentially accumulated in our study overlapped with those identified using a supervised machine learning approach of *N. punctiforme*–*P. schreberi* symbiosis (Warshan et al. 2017), ten proteins were identified in a transcriptomic analysis in response to *A. punctatus* (Campbell et al. 2008) and seven proteins coincided with those identified in a proteomic analysis of freshly isolated *Nostoc* strains from the symbiotic gland tissue of *G. manicata* (Ekman et al. 2006). These proteins are involved in signal reception, adhesion, plant cell wall degradation and response to oxidative stress (some are highlighted in **Fig. 5**). A high number of coincident proteins in three different *Nostoc*–plant symbioses suggest a low host selectivity of this symbiotic cyanobacterium. More than 30 years ago, *N. punctiforme* was isolated from the cycad *Macrozamia communis*, and it is now known that it is involved in intra- and extracellular symbiosis with all four of the major phylogenetic divisions of terrestrial plants (Meeks and Elhai 2002, Adams et al. 2013). The increased abundance of chemotaxis proteins CheY, PtxB and PtxD in *N. punctiforme* in response to *O. sativa* is indicative of a positive reception of the plant partner. At the transcriptional level, induction of these chemoreceptors has been reported in *N. punctiforme* in response to other plant partners such as *A. punctatus, P. schreberi* and *Burmannia pusilla* (Campbell et al. 2008, Duggan et al. 2013, Warshan et al. 2017). This might be indicative that cyanobacteria, unlike rhizobia, have evolved to sense signals from multiple partner representatives of the major phylogenetic groups of plants. Thus, induction of membrane receptors involved in phototaxis and chemotaxis might be essential in early colonization processes and symbiotic competence of *N. punctiforme*.

In symbiotic associations between cyanobacteria and plants, transport of amino acids, sugars and ions occupies a central position (Roy et al. 2020). A recent metatranscriptomic analysis of symbiotic strains of *Nostoc* has shown the importance of membrane transporters in establishing the association with the host and adapting to their environment (Warshan et al. 2018). In our proteomic study, components of different uptake transport systems, mediating the uptake of amino acids, potassium, phosphonates and molybdenum were detected in the cyanobacterium in response to *O. sativa*. We noted that three of the four components of a *kdp-*type K^+^ uptake system (B2J9H1, B2J9H2 and B2J9H4) showed a strong increased abundance in response to the plant partner. In *N. punctiforme*, as it occurs in other bacteria, genes encoding this transporter are arranged in an operon. In bacteria, Kdp is the only bacterial K^+^ uptake system whose expression is strongly regulated at the transcriptional level in media containing low K^+^ concentrations (Epstein 2003). We do not know whether the induction of this transporter suggests the existence of still unexplored osmoadaptation mechanisms. However, a similar effect has been found in *Sinorhizobium meliloti*–*Medicago sativa* symbiosis (Domínguez-Ferreras et al. 2009). We also found a strong induction of an OprB-type cyanobacterial porin (B2IWS8) in response to *O. sativa*. OprB-type porins mediating the transfer of glucose and fructose in the cyanobacterium have been found to be essential to maintain a metabolic flux of sugars in the *Nostoc*–*Anthoceros* symbiosis (Ekman et al. 2013). Our results reinforce this hypothesis, highlighting the relevance of the transfer of saccharides in the pre-symbiotic *Nostoc*–*Oryza* crosstalk.

In both organisms, *N. punctiforme* and *O. sativa*, we have detected a number of proteins involved in cell adhesion and cell wall modification (**Fig. 5**). In *O. sativa*–AM fungi symbiosis, the plant activates the expression of three FLAs (Gutjahr et al. 2015), which are a subclass of AGPs important for plant development, cell adhesion and signaling (Ma and Zhao 2010, Seifert 2018, Wu et al. 2020). Arabinogalactans associated with AGPs are the main components of *Gunnera* mucilage, a potent hormogonium inductor and chemoattractant required for *G. manicata*–*Nostoc* sp. endosymbiosis (Rasmussen et al. 1996, Khamar et al. 2010). Therefore, induction of FLA proteins in *O. sativa* may positively influence the attraction of *N. punctiforme* during early stages of the symbiotic process.

Symbiotic and pathogenic microorganisms of plants induce a similar set of plant signaling components that facilitate colonization (Hentschel et al. 2000, Zeilinger et al. 2016). Three type III peroxidases were overexpressed in *O. sativa* during early symbiotic events with *N. punctiforme* (**Fig. 5**), which is comparable to a previous report of rice–AM fungi symbiosis (Guimil et al. 2005, Gutjahr et al. 2008, 2015). Studies have shown that type III peroxidases react with hydroxyl radicals to detoxify plant roots (Almagro et al. 2009); however, their role in symbiosis is poorly understood. There is some evidence that type III peroxidases are important for plant cell growth by loosening of the cell wall during AM fungi infection (Gutjahr et al. 2008). Interaction with beneficial microbes activates host plant defense mechanisms to facilitate symbiosis. Thus, glutathione S-transferase (GST) enzymes are induced in *O. sativa* in response to AM fungi symbiosis to cope with toxic reactive oxygen species (ROS) and lipid hydroperoxides (Gutjahr et al. 2015). Our proteomic analysis revealed the differential abundance of three GST enzymes in response to *N. punctiforme*. This is consistent with a recent study showing that *N. punctiforme* induces the expression of GST enzymes in *A. thaliana* to reduce toxic ROS and limit the extent of the hypersensitive response (Belton et al. 2021).

Previous studies have revealed that chemical signaling in the plant partner involves the secretion of flavonoids (Cohen and Yamasaki 2000, Cohen et al. 2002) and diacylglycerols (Hashidoko et al. 2019). Plant signals have a strong impact on the biology of the cyanobacterium, inducing conversion of CO_2_-fixing vegetative trichomes to motile hormogonia, which are the plant infection units (Meeks and Elhai 2002). Increased abundance of a flavonoid 3ʹ-monooxygenase in *O. sativa* provides a first evidence of a similar regulation in *N. punctiforme*–*O. sativa* symbiosis. It has been documented that naringenin and naringin induce expression of genes involved in symbiosis in *N. punctiforme* (Cohen and Yamasaki 2000). In addition, a chalcone synthase, catalyzing the production of naringenin chalcone, has been identified in a transcriptomic analysis of *Nostoc*–*Azolla filiculoides* symbiosis (Li et al. 2019).

In other plant–microbe symbioses, flavonoids induce the expression of Nod and mycorrhizal (Myc) factors produced by rhizobia or AM fungi, respectively (Mus et al. 2016). Five putative Nod-like proteins were highly expressed in *N. punctiforme* in response to *O. sativa*. They comprise a putative NodB polysaccharide deacetylase and four putative NodE beta-ketoacyl synthases. *Nostoc* DNA sequences homologous to rhizobial *nod* genes were identified two decades ago by heterologous hybridization, but their sequences were never identified (Rasmussen et al. 1996). Genes encoding Nod proteins identified in our proteomic analysis might be those formerly detected in *Nostoc*. Another polysaccharide deacetylase (B2J7M8) has been previously described as a putative NodB enzyme by homology searches (Gunawardana 2019). However, we did not detect this protein in our proteomic analysis. Future studies will determine the involvement of the other Nod-like proteins in the biosynthesis of cyanobacterial Nod factors and the symbiotic competence. Additionally, six proteins with similarity to NodO, required for infection thread development (Mbengue et al. 2020) are detected in the presence of *O. sativa*. However, this may not be the mode of entry of *N. punctiforme* into plant roots. We observed that the infection started in the plant body, then extended through the root hairs, with the root tips unaffected (**Fig. 1C**; Álvarez et al. 2020).

In legume–*Rhizobium* symbiosis, NFs induce depolarization of the cytoplasmic membrane and calcium spiking response, activating genes of the CSSP pathway (Kosuta et al. 2008). It has been proposed that genes in the CSSP are invariantly conserved in all land plant species possessing intracellular symbionts (Radhakrishnan et al. 2020). However, other studies show that the CSSP is involved in the perception of signals from other microbes beyond the restricted group of endosymbiotic interactions *sensu stricto*, such as the parasitic fungus *Fusarium solani*–*L. japonicus* association (Skiada et al. 2020) and the ectomycorrhizal fungus *Laccaria bicolor*–populus association (Cope et al. 2019). In the *Nostoc*–plant symbioses, in which the same cyanobacterial strain provides both intra- and extracellular associations, it is thought that each group of symbiotic land plant partners evolved different mechanisms, arriving at a stable, competitive symbiotic association (Meeks 1998). Our results provide compelling evidence for the requirement of the CSSP pathway for *N. punctiforme* accommodation into *O. sativa* roots. Further studies are needed to know whether the CSSP is a general mechanism of all *Nostoc*–plant symbioses or is restricted to intracellular symbioses, as is the case for *N. punctiforme* and *O. sativa*.

## Materials and Methods

### Organisms and growth conditions

*Oryza sativa* L. ssp. indica var. Puntal was used for proteomic analysis and characterization of the *N. punctiforme*–*O. sativa* symbiosis. *Oryza sativa* L. ssp. japonica var. Nipponbare background was used for genetic analyses, with homozygous lines mutated in POLLUX (NC6423 and ND5050 for *pollux*-2, and *pollux*-3, respectively), CCaMK (lines NE1115 and NF8513 for *ccamk*-1 and *ccamk*-2, respectively) and CYCLOPS (lines NC2415 and NC2713 for *cyclops*-2 and *cyclops*-3, respectively) (Gutjahr et al. 2008). All *O. sativa* variants were kindly provided by Prof. Uta Paszkowski, University of Cambridge, UK.

Rice seeds were washed with distilled water and then surface sterilized with 5% (w/v) calcium hypochlorite for 20 min. The seeds were thoroughly washed with sterilized tap water and germinated on wet filter paper in a container. Rice plants were grown hydroponically in 50-ml conical tubes with BG11_0_ (-N) medium (free of combined nitrogen). Experiments were carried out in a growth chamber at 28°C, 75% relative humidity, 12-h light–12-h dark cycles, at a light intensity of 50 µmol· m^−2^· s^−1^.

*Nostoc punctiforme* PCC 73102 was obtained from the Institute of Plant Biochemistry and Photosynthesis and grown in BG11_0_ medium supplemented with 17 mM KNO_3_, at 30°C under continuous light (50 µmol· m^−2^· s^−1^) in shaking liquid cultures (100 rpm) or solid media supplemented with 1% w/v agar. To prevent hormogonium differentiation, filaments of *N. punctiforme* were grown to a concentration of 2–3 µg Chl· ml^−1^ in BG11_0_ medium supplemented with 17 mM KNO_3_ and 4 mM sucralose (Splitt and Risser 2016). Induction of hormogonium differentiation was carried out by washing and incubation in BG11_0_ medium without sucralose for 16 h.

### Co-cultivation of *N. punctiforme* and *O. sativa*

Seedlings of *O. sativa* grown for 7 days were suspended in 50-ml conical tubes with BG11_0_ medium. After 4–5 d of acclimation, *N. punctiforme* inoculants containing hormogonia were added to the solutions at a final concentration of 0.8 µg Chl· ml^−1^. Co-cultivation was carried out in a growth chamber for up to 35 d as described above for rice plants. The cultures of each partner were grown separately in parallel as controls. The association of *N. punctiforme* with plant roots was determined by measuring chlorophyll *a* content expressed per root fresh weight as described in Álvarez et al. (2020), according to Arnon (1949).

### Confocal microscopy

Fresh rice roots from plants that had been co-cultured with *N. punctiforme* for 35 d were cut and washed in running tap water. Samples were mounted on a coverslip and examined with a Leica TCS SP2 confocal microscope using HC PL-APO CS ×10 or HCX PLAM-APO ×63 1.4 NA oil immersion objectives. Cyanobacterial autofluorescence was excited at 488 nm from an argon laser, and fluorescence emission was monitored at 650–700 nm. Root lignin autofluorescence was excited with 476 and 488 nm laser irradiation, and fluorescence emission was monitored at 510–533 nm. Z-series slices containing 25–130 frames were stacked and processed using ImageJ program, version 1.41 (Schneider et al. 2012).

### Analysis of colonization

Roots from rice plants co-cultured with *N. punctiforme* for 35 d were used. Loosely attached cyanobacteria were first removed from rice roots by extensive washing with detergent in running tap water. Subsequently, the roots were dried, excised from the plant and weighed. Samples containing 20 mg of fresh weight were then surface sterilized by immersing the plant roots in sterilization solution [1% bleach, 0.1% sodium dodecyl sulfate (SDS) and 0.2% Tween20] for 5 min followed by three washes with sterile water and a final wash with BG11_0_. Lack of bacterial growth in this last wash with BG11_0_ was corroborated by growth tests. Surface-sterilized roots were dissected in small pieces with a sterile scalpel in order to favor the growth of *N. punctiforme* colonizing intracellularly and placed in an Erlenmeyer flask containing 50 ml of BG11_0_ medium supplemented with 17 mM KNO_3_ and 100 µg/ml cycloheximide. Cultures were incubated at 30°C under continuous light (50 µmol· m^−2^· s^−1^) and shaking (100 rpm) for 10 d. After this period, chlorophyll was quantified as previously described.

### Sample preparation and proteomic analysis by SWATH-MS

Three independent biological replicates of fresh hormogonia from *N. punctiforme* and seedlings of *O. sativa* were co-cultured in BG11_0_ to deduce the proteome coverage of the early steps of recognition. Axenic cultures of each species were grown in parallel as the controls. For controls, total protein extracts from three independent biological replicates of *N. punctiforme* (5 ml of culture at 50 µg Chl· ml^−1^ per replicate) and *O. sativa* root samples (300 mg from 15 plants per replicate) were prepared at 1 dpi and 7 dpi. For the inoculated samples, 15 plants per replicate containing *N. punctiforme* attached to the roots were used. Samples were ground in liquid nitrogen, incubated in lysis buffer [50 mM Tris-Cl pH 7.5, 1 mM phenylmethanesulfonylfluoride, 1 mM ethylenediaminetetraacetic acid (EDTA), 2% (w/v) SDS and Complete™ mini EDTA free protease inhibitor cocktail] and centrifuged at 16,000 ×*g* for 15 min at 4°C. Protein concentrations were determined using the Bradford method (Bio-Rad) according to the manufacturer’s instructions. Twenty-microgram protein samples were precipitated by trichloroacetic acid/acetone. The precipitated samples were resuspended in 50 mM ammonium bicarbonate with 0.2% RapiGest (Waters) for protein determination. The resuspended protein samples were digested with trypsin as previously described (Vowinckel et al. 2014). SWATH-MS analysis was performed at the proteomic facility of the Institute of Plant Biochemistry and Photosynthesis, Seville, Spain. A data-dependent acquisition approach using nano-liquid chromatography–mass spectrometry (LC-MS)/MS was initially performed to generate the SWATH-MS spectral libraries.

Peptide and protein identification utilized the ProteinPilot™ software (version 5.0.1, Sciex) with the Paragon™ Algorithm. The search was conducted against the UniProt *O. sativa* proteome, UniProt *N. punctiforme* proteome or a combined database with the UniProt *O. sativa *+ *N. punctiforme* proteome. Automatically generated reports in ProteinPilot were manually inspected for false discovery rate (FDR); cut-off proteins with only proteins identified at an FDR ≤1% were considered for subsequent analyses. Protein-specific peptides and peptides that were not shared between the two organisms were used to generate two species-specific (*O. sativa* or *N. punctiforme*) spectral libraries.

SWATH analysis was quantified using three technical replicates of each biological replicate using a data-independent acquisition method. Each sample (1 μg of protein) was analyzed using the SWATH-MS acquisition method using a LC-MS equipment, AB Sciex, model 5600. The method consisted of repeated acquisition cycles of time-of-flight mass spectrometry (TOF MS)/MS scans (230–1,500 *m*/*z*, 60 ms acquisition time) of 60 overlapping sequential precursor isolation windows of variable width (1 *m*/*z* overlap) covering the 400–1,250 *m*/*z* mass range from a previous TOF MS scan (400–1,250 *m*/*z*, 50 ms acquisition time) for each cycle with 3.7 s total cycle time. Autocalibration of the equipment and chromatographic conditions were controlled by an injection of trypsin-digested β-galactosidase standard from *Escherichia coli* between the replicates.

SWATH-MS spectral alignment was performed using the PeakView 2.2 software (Sciex) with the MicroApp SWATH 2.0 using the *O. sativa* or *N. punctiforme* spectral libraries. After data processing, the processed mrkw files containing protein information were loaded into MarkerView software (version 1.2.1.1, AB Sciex) for normalization of protein intensity (total area sums) using the built-in total ion intensity sum plug-in.

### Computational methods

The quality of the normalized data was analyzed using PCA, and the results were visualized using the ggbiplot R package. Normalized data were then processed using the linear models for microarray data (LIMMA) package in R (Ritchie et al. 2015) to extract changes in protein abundance. We performed comparisons between the cyanobacterial samples in the presence or absence of *O. sativa* at time points of 1 dpi and 7 dpi: *N. punctiforme* plus *O. sativa* after 24 h (*Nostoc_Oryza*_24h) versus *N. punctiforme* alone after 24 h (*Nostoc*_24h), *N. punctiforme* plus *O. sativa* after 7 days (*Nostoc_Oryza*_7d) versus *N. punctiforme* alone after 7 days (*Nostoc*_7d), *N. punctiforme* plus *O. sativa* after 7 days (*Nostoc_Oryza*_7d) versus *N. punctiforme* plus *O. sativa* after 24 h (*Nostoc_Oryza*_24h) and *N. punctiforme* alone after 7 days (*Nostoc*_7d) versus *N. punctiforme* alone after 24 h (*Nostoc*_24h). The same comparisons were used for plant samples. A total of 1,076 cyanobacterial proteins and 736 plant proteins had a statistically differential abundance in at least one comparison (fold change > 1.5 or < 0.66667, and adjusted *P*-value < 0.05). Clustering analysis was carried out using Mfuzz R package (Kumar and Futschik 2007) using a ‘fuzzifier’ of 1.25647 (*m* = 1.25647) for *N. punctiforme* and 1.265078 (*m* = 1.265,078) for *O. sativa*. Cyanobacterial or plant proteins considered to be in the core of a cluster were selected using a strict confidence threshold of 0.80.

### Statistical analyses

The data recorded for the growth, weight and colonization assays were subjected to analysis of variance and t-tests in accordance with the experimental design, using XLMiner Analysis ToolPak in Excel to quantify and evaluate the source of variation. Standard deviations are depicted in the graphs, and statistically significant differences were calculated at a probability of 0.05.

## Supplementary Material

pcac043_Supp

## Data Availability

Mass spectrometry raw proteomic data were deposited in the ProteomeXchange Consortium via the PRIDE partner repository with the identifier PXD022229. Further information of the raw data uploaded to PRIDE is included in Supplementary data File 2. Requests for materials should be addressed to V.M. and C.A.
