# Supplementary material for: Quantitative Proteomics at Early Stages of the Symbiotic Interaction Between Oryza sativa and Nostoc punctiforme Reveals Novel Proteins Involved in the Symbiotic Crosstalk
Source: Plant Cell Physiol. 2022 Apr 4;63(10):1433–45. doi: 10.1093/pcp/pcac043 (PMC9620832; doi:10.1093/pcp/pcac043)
Supplement: pcac043_Supp [file pcac043_supp.zip › pcp-2022-e-00016-File008.pdf]

Supplementary figures and tables

Supplementary Fig. S1. **Effect of *N. punctiforme* inoculation on plant growth and weight.** Rice plants grown in media free of combined nitrogen in the absence (-Np) or presence (+Np) of *N. punctiforme* were photographed and measured at 20 dpi. A) Image of the plants after XX days of co-culture. B) Length of the aerial and radical part of the plants. C) Weight of the aerial and radical part of the plants. The values are the means ± standard error from three independent biological replicates. P indicates P value from the Student's t-test.

Supplementary Fig. S2. Principal component analysis of the data. A) Scatter plots of the first two principal components of the normalised protein expression profiles in *N. punctiforme* at 1 dpi and 7 dpi in the presence and absence of *O. sativa*. **B)** Scatter plots of the first two principal components of the normalised protein expression profiles in *O. sativa* at 1 dpi and 7 dpi in the presence and absence of *N. punctiforme*.

Supplementary Fig. S3. Clustering results of differentially expressed cyanobacterial proteins. The data are plotted as normalised expression changes (Z-score) on the y axes and different conditions on a nonlinear scale on the x axes. The dark line in each plot represents the average of all the genes within that cluster.

Supplementary Fig. S4. Clustering results of differentially expressed *O. sativa* proteins. The data are plotted as normalised expression changes (Z-score) on the y axes and different conditions on a nonlinear scale on the x axes. The dark line in each plot represents the average of all the genes within that cluster.

Supplementary data File 1. Proteins identified and quantified in *N. punctiforme and O. sativa* at 1 and 7 days of co-culture.

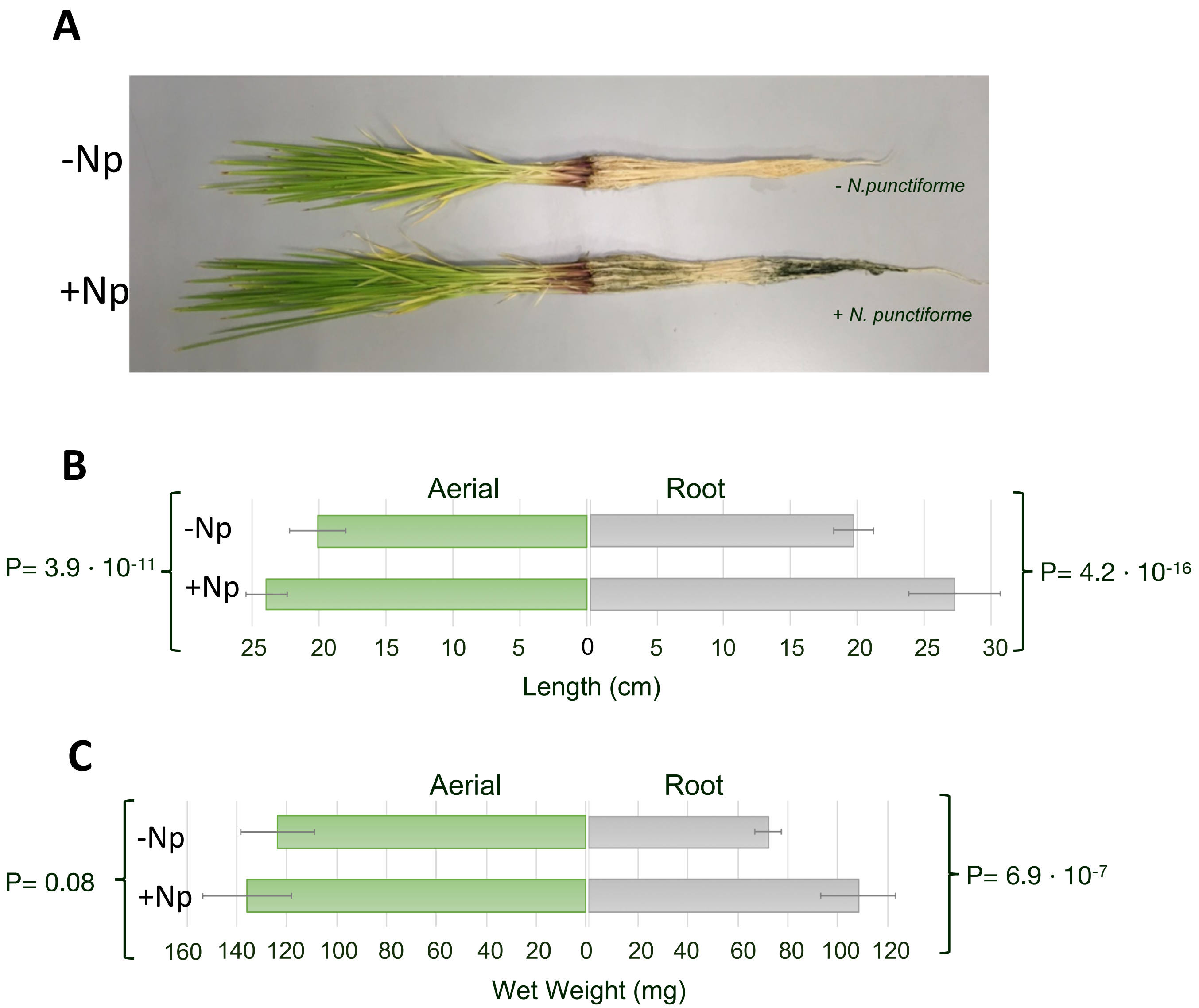

Álvarez et al. Figure S1

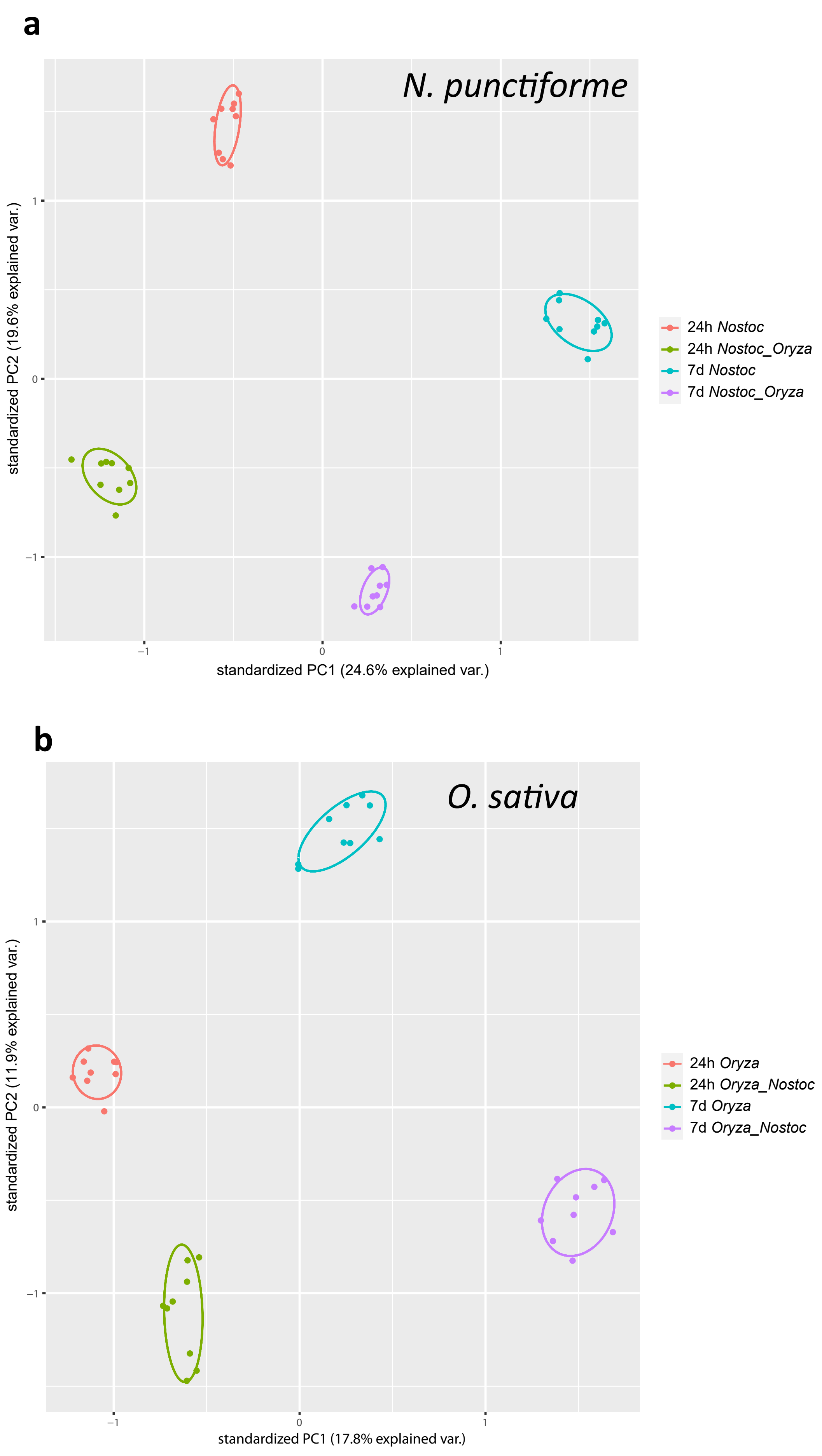

Álvarez et al. Figure S2

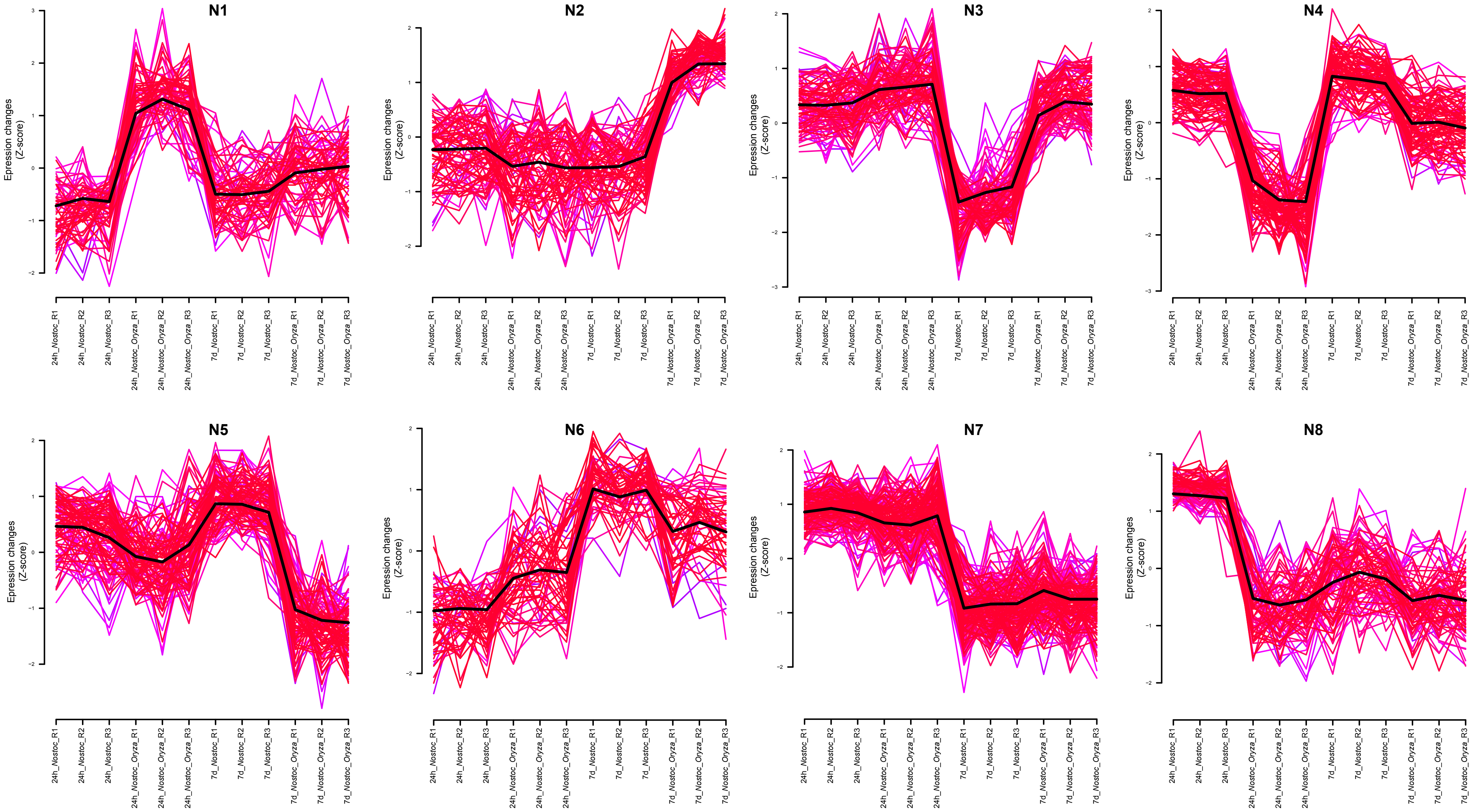
N1
N2
N3
N4
N5
N6
N7
N8
Epression changes
(Z-score)
24h_Nostoc_R1
24h_Nostoc_R2
24h_Nostoc_R3
24h_Nostoc_Oryza_R1
24h_Nostoc_Oryza_R2
24h_Nostoc_Oryza_R3
7d_Nostoc_R1
7d_Nostoc_R2
7d_Nostoc_R3
7d_Nostoc_Oryza_R1
7d_Nostoc_Oryza_R2
7d_Nostoc_Oryza_R3

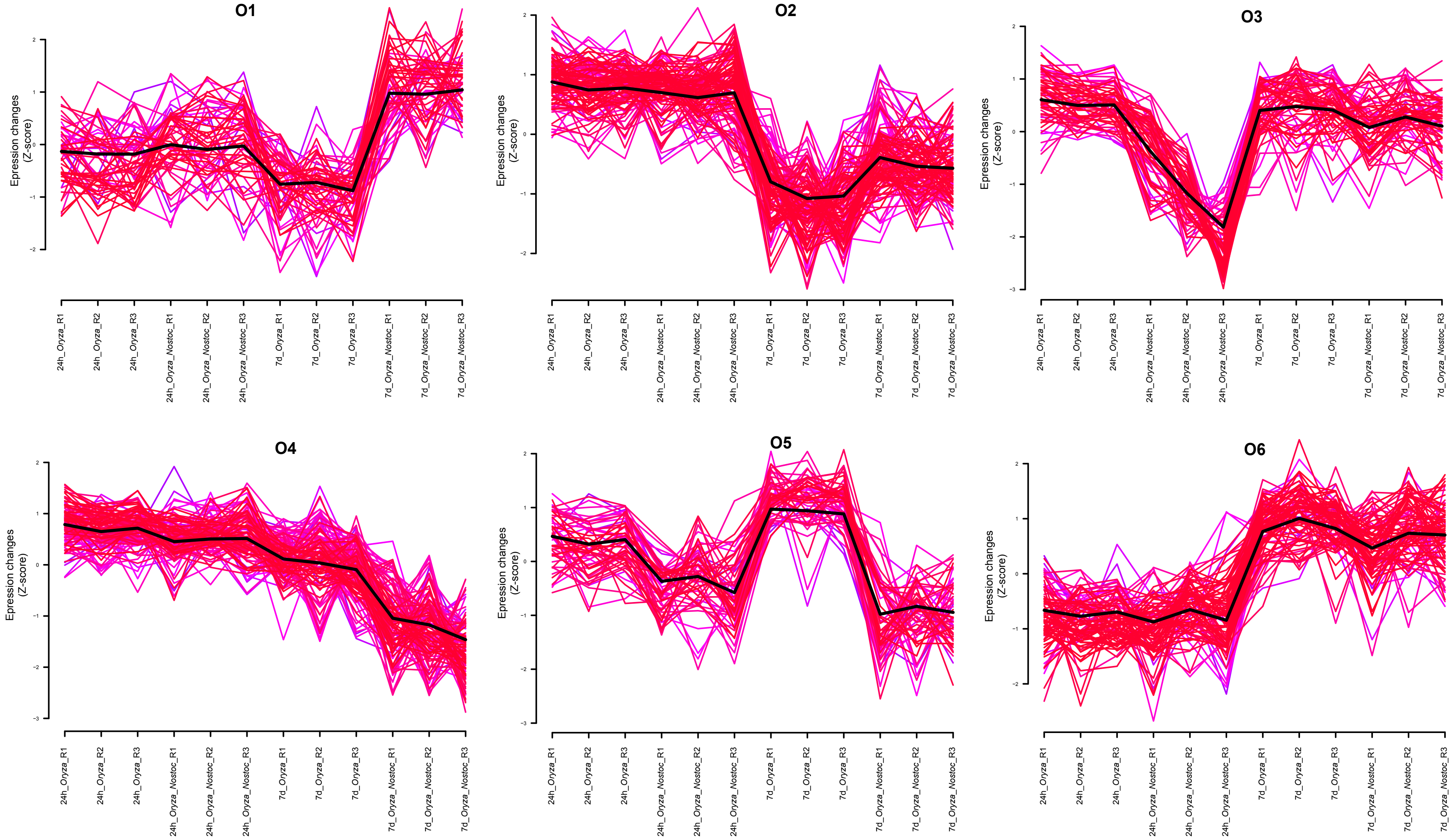
O1
O2
O3
O4
O5
O6
Epression changes
(Z-score)
24h_Oryza_R1
24h_Oryza_R2
24h_Oryza_R3
24h_Oryza_Nostoc_R1
24h_Oryza_Nostoc_R2
24h_Oryza_Nostoc_R3
7d_Oryza_R1
7d_Oryza_R2
7d_Oryza_R3
7d_Oryza_Nostoc_R1
7d_Oryza_Nostoc_R2
7d_Oryza_Nostoc_R3
